# Broadband 200-nm second-harmonic generation in silicon in the telecom band

**DOI:** 10.1038/s41377-020-0254-7

**Published:** 2020-02-06

**Authors:** Neetesh Singh, Manan Raval, Alfonso Ruocco, Michael R. Watts

**Affiliations:** Research Laboratory of Electronics, Massachusetts Institute of Technology, 77 Massachusetts Avenue, Cambridge, MA 02139 USA

**Keywords:** Nonlinear optics, Ultrafast photonics

## Abstract

Silicon is well known for its strong third-order optical nonlinearity, exhibiting efficient supercontinuum and four-wave mixing processes. A strong second-order effect that is naturally inhibited in silicon can also be observed, for example, by electrically breaking the inversion symmetry and quasi-phase matching the pump and the signal. To generate an efficient broadband second-harmonic signal, however, the most promising technique requires matching the group velocities of the pump and the signal. In this work, we utilize dispersion engineering of a silicon waveguide to achieve group velocity matching between the pump and the signal, along with an additional degree of freedom to broaden the second harmonic through the strong third-order nonlinearity. We demonstrate that the strong self-phase modulation and cross-phase modulation in silicon help broaden the second harmonic by 200 nm in the O-band. Furthermore, we show a waveguide design that can be used to generate a second-harmonic signal in the entire near-infrared region. Our work paves the way for various applications, such as efficient and broadband complementary-metal oxide semiconductor based on—chip frequency synthesizers, entangled photon pair generators, and optical parametric oscillators.

## Photonics: velocity matching broadens silicon’s nonlinear possibilities

Advances in silicon engineering now allow waveguides to deliver frequency doubled light across the entire near-infrared spectrum, an important range for biological imaging and optical communication. Second-harmonic generation, which is normally restricted in silicon because of its underlying crystal symmetry, can be observed by applying strong electric field in silicon, however, the spectral response remains quite narrow to a few nanometers. Neetesh Singh from the Massachusetts Institute of Technology, in Cambridge, United States, and colleagues report a very broadband response of 100s of nanometers using silicon waveguides containing rows of diode junctions. When an electric field is applied to the waveguide, the diodes disrupt the typical silicon symmetry and enable second-harmonic generation from a pump laser. Modifying the cross section of the waveguide to ensure that pump and signal pulses travel though the waveguide at similar velocities enabled generation of frequency doubled light with a broader bandwidth than seen with current silicon or any other material based microstructures.

## Introduction

Modern nonlinear optics is considered to have originated in the demonstration of second-harmonic generation (SHG) soon after the invention of the laser by Franken et al. in the 1960s (ref. ^1^). Since then, a plethora of optical nonlinear phenomena have been demonstrated. In particular, second-order optical nonlinearity-based processes are intensively investigated with various platforms and have been instrumental in applications, such as frequency metrology^2–4^, pulse characterization^5^, compression and shaping^6^, laser sources at unconventional wavelengths^7,8^, and the generation of entangled photon pairs^9^. Bulk SHG, however, is limited to materials that do not possess inversion symmetry. Although such materials are currently emerging in integrated platforms^10–16^, for mass production, a complementary-metal oxide semiconductor (CMOS) material is desirable.

Most CMOS materials are either centrosymmetric (semiconductors) or isotropic (oxides) and therefore do not exhibit a bulk second-order susceptibility (*χ*^(2)^), except at interfaces where the inversion symmetry is broken—a fact that is used for sensitive interface and surface characterizations^17–19^. There have been efforts to observe bulk *χ*^(2)^ effects by inducing stress^20–22^ or optical poling^23^ in the material; however, the net conversion efficiency is low either due to a weak *χ*^(2)^ or the lack of phase matching between the signal and the pump. Recently, by employing an electric-field-induced second harmonic^24,25^, and quasi-phase matching (QPM) scheme^26,27^, we demonstrated a strong *χ*^(2)^ susceptibility (41 pm/v) and efficient SHG up to 13%/W at 1.15 µm^28^. The SHG bandwidth in this work, as in most demonstrations with an integrated platform to date, was limited to a few nanometers due to the walk-off between the pump and signal, resulting from the lack of group velocity matching between the pump and the signal. Relatively, broader SHG can be obtained before the interacting waves have walked off from each other by using shorter devices; however, unsurprisingly, the efficiency is reduced significantly.

Achieving broadband SHG with high efficiency is a significant challenge regardless of whether the configuration is free space with bulk optics or a fully integrated photonics platform. In the past three decades, several techniques have been explored. For instance, in the collinear configuration, one of two methods, gradually varying the QPM period^29–31^ or employing group velocity matching between the pump and the signal^32,33^, is employed. A variation in the QPM for broadband operation is quite straightforward, as different wavelength components are generated at different positions along the length of the waveguide; however, this comes at the cost of a reduced conversion efficiency and an undesired lengthening of the SHG pulse in time^6^. The method involving group velocity matching is quite promising, but for a conventional material with a strong type-0 interaction (pump and signal of the same polarization), it is difficult to engineer the dispersion to match the group velocities of the pump and the signal. This difficulty compels researchers to use either a complex geometry of the pulse (tilted pulse front, in a non-collinear interaction)^34^ or QPM period^35^ or match the group velocities with weak type-1 or type-2 interactions^36,37^. Although these reports are impressive, it is difficult or impossible to employ these methods on an integrated platform.

Due to the well-established CMOS foundry and the high refractive index contrast offered by silicon^38^, one can control the group velocities with dispersion engineering of the waveguide for the same or different optical modes of the pump and signal. Furthermore, with group velocity matching, one can investigate the effect of the pump on the second harmonic through the third-order susceptibility (*χ*^(3)^), such as cross-phase modulation (XPM), which, according to our knowledge, has not been explored before. In this work, we utilize not only group velocity matching but also the strong third-order nonlinearity of silicon to generate a second-harmonic signal over a 200 nm bandwidth near the telecom band, which is the broadest SHG observed on an integrated platform. The broadening of the second-harmonic signal occurs mainly due to the broadening of the pump though strong self-phase modulation (SPM) and the simultaneous XPM of the second-harmonic signal by the pump. SHG broadening based on XPM is especially interesting, as it offers broad signal generation on either side (longer or shorter wavelength) of the signal by varying the pump group velocity relative to the signal group velocity. Furthermore, we demonstrate a waveguide design, in which the dispersion can be modified relatively easily for broad group velocity matching, thus supporting SHG over an octave in the near-infrared (IR) region without requiring a corresponding octave-spanning pump.

Such a device will be useful not only for efficient carrier–envelope phase stabilization of a mode-locked laser^39,40^ in a self-referencing scheme by effectively increasing the spectral overlap of the second harmonic and the supercontinuum^41,42^ but also for broadband single-cycle biphotons^43^, mid-IR-to-near-IR wavelength translation^44^, high-frequency ultrashort pulse generation^6^, and optical parametrical oscillators.

## Results

**Fig. 1:**
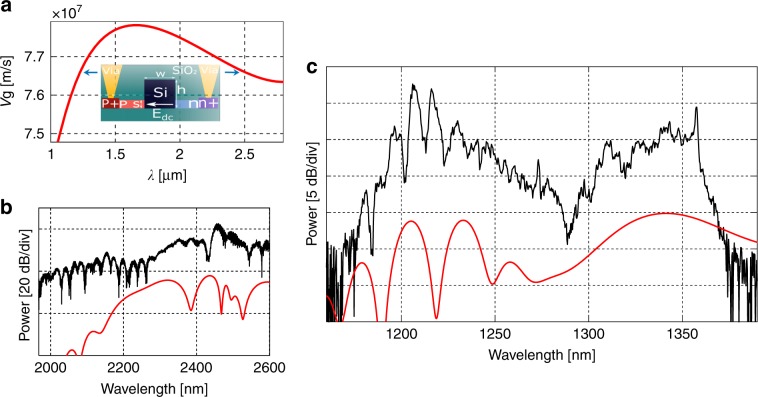
**a** Group velocity curve and waveguide cross section (inset). The arrows indicate the group-velocity-matching wavelength. **b** Experimental (black) and simulated (red) spectra of the supercontinuum at the pump wavelength. **c** Experimental (black) and simulated (red) second-harmonic generation spectra

We designed and fabricated a broadband second-harmonic silicon waveguide in a standard silicon CMOS foundry that was pumped by 200-fs pulses at 2.48 µm with 100 W of coupled peak power (Materials and methods section). To break the centrosymmetry of silicon, a constant electric field was applied across the waveguide through the p-i-n junction using direct current (DC) probes (Quater Research) connected to a voltage supply (Keathley 2231). The output signal was collected by butt coupling a multimode fiber (InF3, Thorlab) to the output facet. The SHG and the supercontinuum at the pump wavelength were measured using two different optical spectrum analyzers (OSAs), Yokogawa AQ6370D and AQ6375B, respectively. The measured supercontinuum generation is shown in Fig. 1b. The supercontinuum is based on the soliton fission process and spans from 1.8 µm to above 2.6 µm. The calculated soliton number is 13, and the soliton fission length is ~2.4 mm. By applying a DC electric field across the waveguide with a voltage of −27 V, a second-harmonic signal spanning 1170–1370 nm was generated, as shown in Fig. 1c. The observed peaks of the supercontinuum and the second harmonic spectra are −53 dBm/nm (at 2.455 µm) and −67 dBm/nm (at 1.206 µm), respectively. The maximum measured SHG conversion efficiency with 200-fs pulses is 112%/W^45^. The SHG spectrum has two large lobes, one at ~1350 nm and the other at ~1210 nm (with overlapping smaller lobes), the origin of which is the combination of the strong SPM of the pump and the XPM of the second harmonic by the pump, which is moving slightly slower than the signal.

For a further investigation, simulations of the second-harmonic and supercontinuum processes were performed using coupled mode equations, similar to those used in ref. ^46^ and modified here for a silicon waveguide.

1 $$\begin{array}{l}\frac{{\partial E_p}}{{\partial z}} = - \left( {\beta _{1p} - \beta _{1s}} \right)\frac{{\partial E_p}}{{\partial t}} - j\mathop {\sum }\limits_{n = 2}^8 \frac{{j^n\beta _{np}}}{{n!}}\frac{{\partial ^nE_p}}{{\partial t^n}} - j\gamma _p\left( {\left| {E_p} \right|^2 + 2\left| {E_s} \right|^2} \right)E_p\\ - jK_pE_p^ \ast E_s{\mathrm{exp}}\left[ { - j\delta k_oz} \right] - \left( {\frac{{\alpha _p}}{2} + 3pa\left| {E_p} \right|^4} \right)E_p\end{array}$$

2 $$\begin{array}{l}\frac{{\partial E_s}}{{\partial z}} = - j\mathop {\sum }\limits_{n = 2}^6 \frac{{j^n\beta _{ns}}}{{n!}}\frac{{\partial ^nE_s}}{{\partial t^n}} - j\gamma _s\left( {\left| {E_s} \right|^2 + 2\left| {E_p} \right|^2} \right)E_s\\ - jK_sE_p^2\exp \left[ {j\delta k_oz} \right] - \left( {\frac{{\alpha _s}}{2} + 2pa\left| {E_s} \right|^2} \right)E_s\end{array}$$

Here, *Ep* and *Es* are the pump and the second-harmonic field envelopes, respectively. *β*_1*p*,*s*_ is the first-order dispersion related to the group velocity as *v*_gs_ = *1*/*β*_1*p*,*s*_. For completeness, up to eight- and sixth-order dispersion terms are added to the *Ep* and *Es* evolution equations, respectively. We have not included the nonlinear free-carrier-based absorption and dispersion, because the pump and SH pulse are short in time, thus having negligible temporal overlap with the free carriers, and the pulse repetition time (>12.5 ns) is longer than the free-carrier life time in silicon, especially when the carrier sweep is applied through a reverse bias^47^. Equations (1) and (2) were solved using the split-step Fourier and Runge–Kutta method with nonlinear and dispersion terms solved in the time and frequency domains, respectively.

The third-order nonlinearity (*χ*^(3)^) is included with *γ*_*p*_ and *γ*_*s*_, where *γ*_*p*_ is responsible for the supercontinuum generation around the pump through the Kerr effect, as shown in Fig. 1b. *K*_*p*,*s*_ is the coupling coefficient^28,48^ extracted from the effective *χ*^(2)^ (=3*E*_dc_*χ*^(3)^) in silicon^28,49,50^ of 41 pm/V. *δk*_*o*_ is a constant phase mismatch term, corresponding to the zeroth-order term in the Taylor expansion of the general phase matching condition between the pump and the signal^36,51^, which is given as *δk*_*o*_ = *k*_2*f*_ − 2*k*_*f*_ − 2π/Λ, where *k*_2*f*,*f*_ is the wavevector in the waveguide at the second-harmonic and fundamental wavelengths, and Λ is the QPM period. By varying *δk*_*o*_ by 300 m^−1^ in the simulation and the experiment (not shown), the overall spectral shape changes negligibly, but the signal strength varies within 3–4 dB. The three-photon absorption, two-photon absorption, *α*_*p*_, and *α*_*s*_ account for the nonlinear losses and linear losses for the pump and second harmonic, respectively^52,53^.

The terms proportional to *γ*_*p*_ and *γ*_*s*_ are due to the SPM and the XPM. The SPM causes spectral broadening around the pump wavelength, generating a supercontinuum, as mentioned before, which in turn generates a broad second-harmonic signal at ~1240 nm. The XPM of the second-harmonic signal by the pump generates a signal at ~1350 nm when the pump is moving slightly slower, i.e., ~30 nm/ps, than the second harmonic, as confirmed by the simulation. By forcing the pump and the signal to move at exactly the same velocity in the simulation, the spectral broadening of the signal at ~1350 nm is suppressed, and the small lobes at ~1210 nm disappear (more detail is given below). We note that the SPM and XPM effects by the second harmonic are negligible as the second-harmonic power is low.

**Fig. 2:**
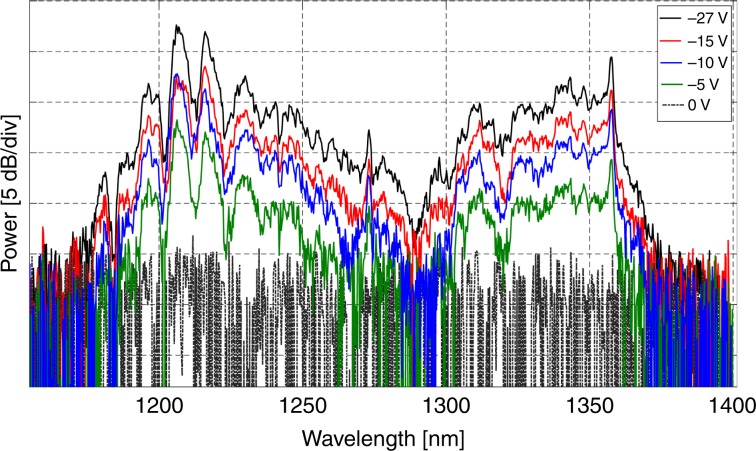
SHG as a function of the reverse-biased voltage

**Fig. 3:**
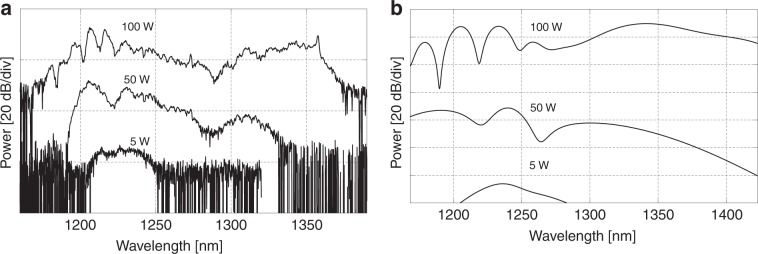
**a**, **b** Experimental and simulated SHG with different pump peak powers

The SHG as a function of the applied DC voltage is shown in Fig. 2. The signal strength increases with increasing applied voltage, while the overall spectral shape is maintained, which is also seen in the simulations (not shown). This result is due to the fact that the applied DC field only changes the nonlinearity factors as *χ*^(2)^=3*E*_dc_*χ*^(3)^. We also measured the SHG response as a function of pump power by using a variable neutral density filter in the beam path of the pump. The power before the coupling lens was measured with an integrating sphere (Thorlab) with the fluctuation being within 1%. The results are shown in Fig. 3 for different pump peak powers (100 W, 50 W, and 5 W). Here, we observe that the long-side lobe (1350 nm) is strongly power-dependent compared to the lobe at ~1210 nm, which reaffirms the XPM origin of the signal at ~1350 nm, since the XPM is twice as sensitive to the pump power as the SPM^54^.

**Fig. 4:**
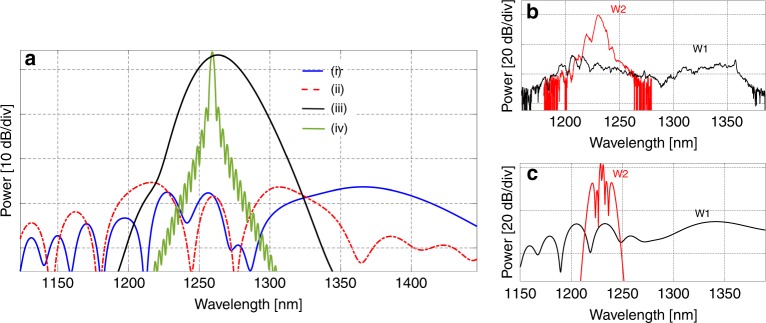
**a** Simulated SHG spectra. (i) The pump and the signal have a small group velocity mismatch (ii) and no group velocity mismatch. (iii) The self-phase modulation of the pump and the cross-phase modulation of the signal are turned off in the simulation, and (iv) a very high group velocity mismatch is employed. **b**, **c** Experimental and simulated SHG spectra for two different waveguides

In the following, we analyze different mechanisms responsible for the spectral broadening of the second-harmonic signal in more detail. In Fig. 4a, several simulated plots are shown, where we used a pump power of 100 W, pump wavelength of 2.52 µm, pulse width of 250 fs, waveguide length of 5 mm, fixed *δk*_*o*_, and dispersion and loss as above, and all the spectra were normalized to a fixed power. Plot (i) represents the situation when the group velocity of the pump is slightly slower than that of the signal, similar to the experiment in Fig. 1c. In this case, there is a strong XPM of the second-harmonic signal by the pump, causing broadening in the long-wavelength region. When the group velocities are perfectly matched, the SHG spectrum looks more symmetric (plot (ii)). In this case, the SPM (of the pump)-based broadening is more prominent, and the long-side broadening aided by the XPM (as in (i)) is weak. This effect occurs because the SPM of the pump broadens the second harmonic almost symmetrically, and since the pump is moving with exactly the same velocity as the signal, the XPM, which is strong only when the signal temporally overlaps with either the rising or the falling edge of the pump pulse, is suppressed, which has been well studied in *χ*^(3)^-based supercontinuum processes^54,55^. For plot (iii), the SPM of the pump and the XPM of the signal were turned off in the simulation, causing a reduction of the spectral broadening of the SHG to a bandwidth determined only by the group-velocity-matching bandwidth that can be achieved experimentally with a tunable continuous wave laser avoiding *χ*^(3)^ effects. Plot (iv) uses similar simulation parameters as plot (iii) except that the group velocity is highly mismatched (by 600 nm/ps) between the pump and the signal. In such a case, the typical sinc-type SHG spectrum appears (as higher-order terms are now relatively weaker)^56^. We must note that the fringes of the side lobes become stronger with a lower-loss waveguide. The small difference in the amplitude of the peaks of plots (iii) and (iv) is due to using a group velocity mismatch of ~30 nm/ps in (iii). The signal peak in (iii) and (iv) is significantly higher than that in (i) and (ii), simply because the SHG power in (iii) and (iv) does not spread over a broad bandwidth.

To experimentally observe the effects of a high group velocity mismatch, and weak SPM and XPM of the pump and signal, respectively, on the SHG, we measured SHG from a waveguide with a long input pulse and large group velocity mismatch between the pump and the signal. The waveguide (W2) has a width of 800 nm, a total height of 380 nm, and a slab thickness of 100 nm. The pump with a peak power of 30 W at 5 ps is launched in the normal dispersion region at ~2.47 µm, thus causing weak SPM-based spectral broadening at the pump wavelength. The result is shown in Fig. 4b (Fig. 4c shows the simulation result), where W1 represents the results in Fig. 1c. The group velocity mismatch of the W2 waveguide is estimated to be a factor of 5 higher than that of W1. As expected, the signal of W2 is less broad than that of W1, which looks similar to but not quite the same as the plot (iv) of Fig. 4a because of using a higher (20×) group velocity mismatch in plot (iv) of Fig. 4a.

**Fig. 5:**
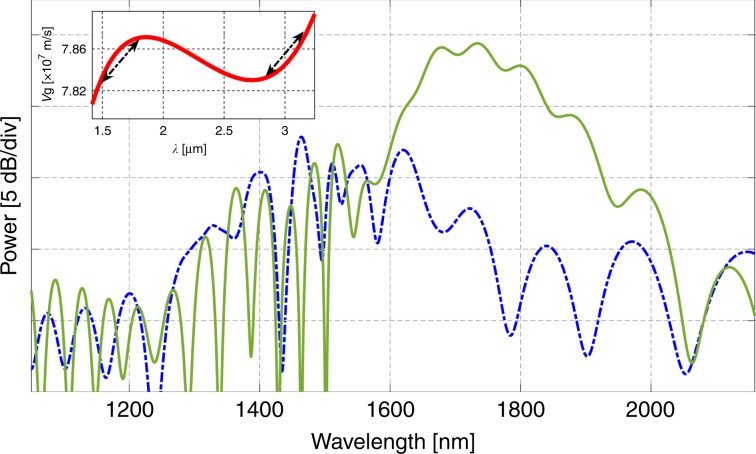
Simulated SHG spectrum with zero (dash blue) and small (solid green) group velocity mismatches. In the group velocity curve (inset), the arrows represent the matching group velocity regions

By increasing the low group velocity mismatch wavelength range between the pump and the signal, such that the group velocity dispersion terms are matched, i.e., 2GVD_2*f*_ − GVD_*f*_ = 0, the SHG bandwidth can be further increased. To show this result numerically, a waveguide was designed with a slab width of 90 nm, waveguide width of 1035 nm, and total height of 380 nm. The simulated SHG bandwidth along with the group velocity curve is shown in Fig. 5. The pump parameters used in the simulations are similar to those in Fig. 1, except that the pump wavelength is centered at 3.2 µm and the peak power is ~200 W. Since the third-order nonlinearity (*χ*^(3)^) of silicon does not vary significantly from 2.5 µm to 3 µm (ref. ^52^), the effective *χ*^(2)^ is considered to be the same as that at ~2.5 µm. With exact group velocity matching between the pump and the signal, SPM-type SHG can be observed. However, with a slight group velocity mismatch (slower pump), the strong XPM of the signal causes strong long-side signal generation. The signal becomes stronger at ~1.7 µm and extends up to 2 µm without having to use a pump at 4 µm, which is beneficial since at 4 µm, significant material loss from the silica substrate is present. The SHG broadening can be further increased to cover the entire near-IR window with higher pump power.

## Discussion

In Fig. 4b, c, we observe that there is a trade-off between the signal broadening and strength; however, there are several disadvantages of using a device like W2. First, the weak SPM of the pump will be of limited use for applications, such as self-referencing, where an octave-spanning supercontinuum is required. Second, longer pump pulses in the anomalous dispersion region tend to be affected by noise-based modulation instability, deteriorating the coherence of the resulting signal required for many applications discussed above^57^. With a device like W1 and a short pump pulse, the coherence is maintained, and XPM can be used to selectively enhance the long or short side of the second harmonic depending on the relative velocity between the pump and the signal. Additionally, the broader SHG can increase the carrier–envelope offset frequency (*f*_ceo_) signal strength for photodetection, as a broader second-harmonic signal can overlap with a broader SC signal, which will be useful for applications in which strong *f*_ceo_ stabilization through a feedback loop is necessary.

In conclusion, in this work, we have characterized broadband SHG in silicon with short pulses. Due to the group velocity matching of the second harmonic with the pump, broadband signal generation up to 200 nm is observed. This is achieved due to the combination of the strong third-order-nonlinearity-based spectral broadening of the pump and the XPM of the second-harmonic signal. XPM-based broadening is especially interesting, as the SHG bandwidth can be selectively increased on the long or short side of the signal depending on the relative velocity between the pump and the signal. Moreover, silicon waveguides can be designed to generate a second-harmonic signal over the entire near-IR region when pumped at ~3 µm. We believe this work is important not only for various photonic systems leveraging the CMOS foundry, but also for fundamental research investigating the effects of a third-order nonlinearity on second-order nonlinear effects in general.

## Materials and methods

A 5-mm-long silicon rib waveguide was designed with a width (w) of 900 nm, total height (h) of 380 nm and slab thickness (s) of 65 nm, as shown in Fig. 1a (inset). The group velocity at the pump (2.48 µm) and signal (1.24 µm) wavelengths is ~7 × 10^7^ m/s. The waveguide was fabricated in a standard 300-mm-line CMOS foundry (CNSE SUNY polytechnic). Dopants were ion implanted in silicon to create p- and n-type regions for applying a reverse bias across the transverse direction of the waveguide for the optical quasi-transverse electric (TE) mode^28^. A row of p-i-n junctions was fabricated along the length of the waveguide with a period of 1800 nm for efficient QPM of the second-harmonic signal with the pump. The presence of carriers (holes and electrons), however, increases the propagation loss of the pump due to the modal overlap with the dopants. The propagation losses at the pump and signal wavelengths are measured to be 6 dB/cm and <1 dB/cm, respectively^45^. Accordingly, a small undoped section of the waveguide at the input was added for efficient spectral broadening of the pump though SPM, which further propagates down into the doped waveguide region. Optimized inverse tapers were used at the input and output ends for efficient coupling. TE-polarized pulses were injected into the waveguide at 2.48 µm from an optical parametric oscillator (Coherent) with a pulse width of 200 fs and a repetition rate of 80 MHz. The pump was coupled to the waveguide with a chalcogenide aspheric lens (Black Diamond-2) with a numerical aperture of 0.5. The coupling to the waveguide was optimized by capturing a streak of light scattered out along the length of the waveguide using an IR camera mounted above the chip. The estimated coupled peak power in the waveguide was ~100 W.
